# The Existential Risks of Nuclear Weapons: A Role for Ethics and Emotions

**DOI:** 10.1007/s13347-026-01097-2

**Published:** 2026-05-08

**Authors:** Sabine Roeser

**Affiliations:** Ethics and Philosophy of Technology Section, Department of Values, Technology and Innovation, Faculty of Technology, Policy and Management, Delft University of Technology, Jaffalaan 5 2628 BX, Delft, The Netherlands

**Keywords:** Nuclear weapons, Risk, Ethics, Emotions, Geopolitics, War

## Introduction

The risk of nuclear weapons is an important topic that does not get sufficient attention in the philosophy of technology. There are and have been some discussions about the ethics of nuclear weapons, but most of them are from non-philosophers. Indeed, it is surprising —and worrisome—that the discussion of nuclear weapons in the philosophy of technology is virtually nonexistent. A noteworthy recent exception is Haiden (2026). In this contribution, I will reflect on some of the topics brought up in Haiden’s important contribution. I will also highlight some additional ethical issues related to nuclear weapons, specifically related to the context of existential risk and the role of moral emotions.

## Ethical Conundrums of Nuclear Weapons

The threat of nuclear war is one that we have to be aware of in order to act on it, but we also need to simultaneously ignore to be able to continue living our lives. In that sense, the risk of nuclear weapons is similar to other existential risks. That in and of itself is a huge existential struggle asking for cognitive dissonance, just as concerning other existential risks, such as related to climate change and Artificial Intelligence (AI). In our daily lives, we need to act as if these risks do not exist, because if we accept that they are there and that they are urgent, this will overshadow everything else we do and threaten to make these other activities appear pointless. Indeed, ignoring the risk of nuclear war is how we have largely dealt with it for decades, but given its lingering presence we should make a continuous effort to also be aware of its existence. At the height of the Cold War, the threat of nuclear war was at the forefront of public debates, but as the geopolitical tensions of the Cold War faded, so too did our focus on it. In contemporary political discussion, nuclear war is not a key topic, despite its ever-present danger. However, even during those past decades, the risk of, for example, an unintended, accidental nuclear launch has been ever-present, and could ignite a full-fledged nuclear war even in a theoretical situation of perfect global peace. Of course, the world has never been in such a peaceful state, and in recent years, rising geopolitical tensions should correspond to an increased awareness of the looming risks of nuclear war. Also, recently the US-Russia nuclear weapons treaty START (New Strategic Arms Reduction Treaty) has expired, while the world faces several wars also involving nuclear powers. This is why Haiden’s article is very important, as it can hopefully help to spark the discussion on ethical challenges of nuclear weapons in philosophy of technology.

In his contribution, Haiden discusses the work of Kenneth Waltz who has been a leading voice in international relations theory arguing that nuclear weapons positively contribute to peace. Crucial to the development of nuclear weapons is nuclear deterrence. During the Cold War, the US and the Soviet Union were locked in an arms race, aimed to keep a relative balance between the main nuclear superpowers, ensuring no power vacuum emerged. At the heart of this idea of deterrence was the so-called MAD doctrine, referring to the idea of mutually assured destruction. In other words, given the fact that several nations have nuclear weapons, starting a nuclear war would be pointless, as a counterattack is guaranteed. The ensuing escalation would inevitably lead to the destruction of at least the countries involved and presumably of all of humanity, given how geopolitically intertwined all countries are with the leading nuclear powers, and also given the unprecedented destructive force of and fallout from nuclear weapons. A possible nuclear war cannot be limited, but is all-encompassing and would lead to a largely uninhabitable planet (cf. Jacobsen, 2024). The idea behind MAD is that the existence of nuclear weapons paradoxically means that they will never be used. Waltz and others have argued that because of this, nuclear weapons have actually brought us peace (Waltz, 1981).

Haiden provides for a very interesting discussion on how philosophy of technology can contribute to understanding the role of nuclear weapons, by drawing on leading ideas of the value ladenness of technology in philosophy of technology (by drawing on e.g. Verbeek, 2006). I think his arguments are very convincing. The very way nuclear weapons are designed gives rise to unique ethical conundrums. What I would like to add in what follows is that an extreme form of hyperrationality and at most a very limited understanding of emotions is crucial to some of the ethical challenges of nuclear weapons. These challenges can be highlighted by focusing on moral emotions.

Central to the principle of nuclear deterrence is the threat that if your country is attacked by nuclear weapons, you will launch a nuclear weapon in retaliation, leading to an escalation that inevitably leads to total mutual destruction—and will never take place for this reason. Another reading of this is that your country will not launch nuclear weapons *unless* you are attacked, meaning that no one will ever launch a nuclear weapon as there is no ‘prime mover.’ A nuclear attack is then a necessary but not yet sufficient condition of launching back. It is likely a psychological necessity to hold on to the idea that a nuclear attack is a sufficient condition. But if an attack really happens, this may need to be reconsidered, because what is the point at that moment, given that mutual nuclear war will result in mutual destruction? Hence, decision-making about the use of nuclear weapons involves contemplating the moves and counter moves of one’s opponent in a hyperrational, chess-like manner. This is also exemplified in game theory, which played a major role during the nuclear arms race and the development of the MAD doctrine.^1^ However, in the urgency of an acute nuclear attack, which requires that decisions be made within minutes, it cannot be ruled out that emotional concerns about the future of humanity tip the balance in the other direction and that leaders of the country under attack accept defeat and choose to limit destruction rather than contribute to the inevitable total destruction of the world as we know it. Such a choice is not ruled out, meaning that the MAD doctrine is not infallible in practice. At the same time, this option cannot be officially mentioned, as it would undermine the MAD doctrine and the stability it promises.

Also, there are risks of the MAD doctrine. For example, a nuclear war can begin unintentionally, as almost happened several times in history. In his book *Command and Control*, Eric Schlosser (2014) discusses the numerous near accidents with nuclear weapons occurring since their inception and around the world. Another scenario can be that a leader in mental or power decline is not concerned about what comes next and could for that reason lead the world down the path of complete destruction. This is the scenario in the book *Nuclear War* by Annie Jacobsen (2024) where a fictitious (and totally destructive) nuclear war is started by a leader who lost his wits.

Haiden discusses Kenneth Waltz’s arguments for the thesis that nuclear weapons supposedly cause peace. Let me reflect on Waltz’s argument. Even if we might grant that nuclear weapons may deter from intentional nuclear war, given the many wars that still take place around the world, also involving nuclear powers, we know that nuclear weapons do not deter from war with conventional weapons. Indeed, while Waltz’s claim may hold for war *between* states having nuclear weapons, we have seen wars by nuclear powers attacking non-nuclear powers ever since WWII. Europe has experienced a long period of relative peace, but we should not forget about the Cold War and the continuous threat that nuclear war formed during that period for the whole world, but also for European countries, that were the potential battleground in case of a nuclear war between the superpowers. There were also numerous non-nuclear wars and interferences taking place in other countries that have been sponsored or lead by the superpowers. The power imbalance between nuclear and non-nuclear powers is a price that non-nuclear powers must pay for a relative power balance between nuclear powers. However, for many countries, living in peace is not a reality. And although a war between e.g., the US and Russia would also have devastating consequences for other countries, it is rather lopsided to ignore the ongoing realities of war for many countries around the globe. Consideration of these issues provides important nuance to Waltz’s claims that seem to be written primarily from the point of view of nuclear superpowers. The reality of war has been ever-present for many people across the world despite decades of nuclear armament. Waltz and others who have made this argument are selective in their choice of evidence. Haiden discusses Waltz’s reflections on the Kargil War between India and Pakistan. Both are nuclear powers, but Waltz argues that the risk of escalation of such clashes is very small because of nuclear weapons (Waltz & Fearon, 2012 as discussed in Haiden, 2026). Nevertheless, a conventional war still broke out, and we can only speculate as to what would have happened had the countries not possessed nuclear weapons.

Haiden also discusses Waltz’s argument against disarmament according to which we cannot trust the information provided to us by other countries and might be betrayed (Waltz & Fearon, 2012). However, this is a peculiar argument: why assume that states would only be dishonest in the case of disarmament and not in the case of a continued arms race or a stable distribution of nuclear arms? On the contrary, in case of joint disarmament, there would presumably be mutual arrangements for inspections, etc.

Furthermore, there are challenges for democracy posed by nuclear weapons. Several authors have argued that nations with nuclear weapons require strict top-down governance systems that are at odds with democratic principles and international law (cf. e.g. Scarry, 2014). Various scholars have pointed out the colonial and anti-feminist aspects of nuclear armament (cf. Choi & Eshle, 2022).

The omnipresent threat of nuclear war can be seen as one of the most pressing existential risks, one that can manifest within a few hours. This threat would hold even in ideal and unrealistic circumstances of complete and everlasting world peace, as a nuclear war can be triggered unintentionally. Even if this would—against all odds and against the premises of the MAD doctrine—not lead to complete nuclear war, the unintended explosion of even one nuclear weapon has more devastating impacts than any other technological accident. And given that we are far away from such an ideal peaceful state, geopolitical tensions make nuclear catastrophes, even if triggered by unintended accidents, even more realistic. The history of near accidents highlights that humanity so far has been extremely lucky to not have been extinguished by an unintentionally triggered nuclear war. This is mainly due to the failure of technologies to work properly (Schlosser, 2014), or because people interpreted a sign of a possible nuclear attack as the result of e.g. a technological glitch.^2^ The decisions of political leaders in crisis situations (e.g., Cuban Missile Crisis) and regarding disarmament (e.g., the role of Gorbachev during the 1980s; cf. e.g. McMahon, 2021) have also prevented nuclear war up to this point.

Nuclear war can be seen as the ultimate reductio of self-interested, macho, competitive policies. Realpolitik is dangerous and self-defeating. In that respect, the MAD doctrine is indeed more ‘mad’ than rational, ironically due to its hyperrational focus. In other words, it is an example of a self-defeating and limited understanding of rationality. A nuclear arms race can be seen as a waste of resources and puts the whole world at risk forever. How can we strive for different politics? Pragmatic idealism and diplomacy are needed to remove root causes of conflict instead of taking them for granted. I would argue that this requires compassion, empathy, and listening to each other (cf. Roeser, 2011, 2018 for my arguments for the role of emotions for ethical decision-making, also in the context of risky technologies). Given these issues, fewer nuclear weapons are arguably better than more, especially because there is already a multitude of the number of nuclear weapons in existence to devastate humanity and destroy the world we know. The resources invested in nuclear weapons could also be spent on other things, e.g., improving the living conditions of people around the world, thereby also contributing to geopolitical stability.

The complex moral conundrums that arise from the existence of nuclear weapons indicate that there are moral dilemmas built into their very design. It is the specific nature and unique ethical features of nuclear weapons that distinguish them from conventional weapons. Nuclear weapons can be seen as a tragic culmination of scientific and engineering ingenuity that can, ironically, lead to the total destruction of the ingenious minds behind them, together with all of humanity. Instead, we should reconceptualize engineering as always serving humanity, based on an attitude of care and compassion (cf. Roeser, 2012). This should be a fundamental criterion for collaborations and projects taken up by engineers and STEM researchers. Emotions can highlight the ethical shortcomings of hyperrational and lopsided approaches such as MAD, an arms race, and the idea that nuclear weapons bring peace. Emotions of care and compassion and feelings of responsibility can lead to different, morally more responsible approaches by politicians as well as by the designers of nuclear weapons.

## Conclusion

That the omnipresent existential risk of nuclear war is not more prominent in public and philosophical debates is psychologically understandable, as it is a horrifying scenario with intricate conundrums that are difficult to solve. Yet, it is the role of ethicists to also bring such unpopular issues to people’s attention. Feelings of responsibility and care can play an important role in doing this. And I am glad that Haiden has started the discussion in our field; I hope that many philosophers of technology will join and help thinking about how we can structurally diminish the risks of nuclear weapons.

## Data Availability

Not applicable.

## Footnotes

1 Cf. e.g. https://science.howstuffworks.com/game-theory5.htm.

2 Cf. e.g. https://en.wikipedia.org/wiki/1983_Soviet_nuclear_false_alarm_incident and https://en.wikipedia.org/wiki/Nuclear_close_calls [accessed 5 April 2026].
